# Concatenated Constrained Coding: A New Approach to Efficient Constant-Weight Codes

**DOI:** 10.3390/e28010078

**Published:** 2026-01-09

**Authors:** Kees Schouhamer Immink, Jos H. Weber, Tuan Thanh Nguyen, Kui Cai

**Affiliations:** 1 Turing Machines Inc., Willemskade 15, 3016 DK Rotterdam, The Netherlands; 2 Department of Applied Mathematics, Delft University of Technology, 2628 CD Delft, The Netherlands; j.h.weber@tudelft.nl; 3 Science, Mathematics and Technology Cluster, Singapore University of Technology and Design (SUTD), 8 Somapah Rd, Singapore 487372, Singapore; tuanthanh_nguyen@sutd.edu.sg (T.T.N.); cai_kui@sutd.edu.sg (K.C.)

**Keywords:** balanced code, concatenated constrained code, constant-weight code, constrained code, Knuth’s algorithm, low-weight code, m-out-of-n code

## Abstract

The design of low-complexity and efficient constrained codes has been a major research item for many years. This paper reports on a versatile method named concatenated constrained codes for designing efficient fixed-length constrained codes with small complexity. A concatenated constrained code comprises two (or more) cooperating constrained codes of low complexity enabling long constrained codes that are not practically feasible with prior art methods. We apply the concatenated coding approach to two case studies, namely the design of constant-weight and low-weight codes. In a binary constant-weight code, each codeword has the same number, *w*, of 1’s, where *w* is called the weight of a codeword. We specifically focus on the trading between coder complexity and redundancy.

## 1. Introduction

A constrained channel is not capable to transmit all possible signals, and only certain sequences may be allowed [1]. A constrained code implements these constraints by converting arbitrary source data into permitted signals [2,3,4]. Designing an invertible mapping from arbitrary, unconstrained source sequences into coded, constrained binary sequences is a fundamental challenge. In a conventional block code, the source data are segmented into small, manageable data blocks, which are then translated into sequences of permitted symbols. The resulting codewords satisfy the given constraints, allowing them to be freely cascaded without violating channel constraints. Naturally, larger block sizes enable more efficient encoding into permitted sequences. Numerous implementation strategies have been explored in the literature.

Implementations fall in three main categories: table look-up, replacement techniques, and arithmetic-based implementations. Table look-up is straightforward, but the size of the data blocks is limited by the maximum size of the tables used [5,6]. The replacement technique successively or iteratively removes forbidden subsequences in the source data to obtain a constrained sequence. Enumeration techniques [4,7,8,9,10,11,12] use integer arithmetic operations to translate source data into constrained codewords and *vice versa*. However, the required silicon area of the look-up table of integer coefficients does not scale linearly with the block size [13], and it may become unacceptably large with mounting word length, precluding their use in practical transmission or storage systems [5]. A second important consideration in enumerative encoding is *error propagation*, as a single bit error in the received codeword may corrupt an entire decoded word [14]. This necessitates the use of strong error correction codes, which in turn require additional redundancy.

Concatenated error correction codes [15] are widely used in data transmission and storage systems. Formed by combining two or more codes, typically referred to as the *inner* and *outer codes*, these constructions provide enhanced error performance while maintaining low decoder complexity. Inspired by this principle, we propose a concatenated constrained coding approach, in which two or more low-complexity constrained codes are combined to construct long constrained codes that are infeasible using conventional methods.

In the encoding device, the source data blocks are divided into two segments: a first segment and a second segment. The first segment of the source data is further partitioned into a plurality of small data subwords that are translated into an allowed codeword using several look-up tables whose sets of constrained output words are mutually disjoint. The second segment of source data is encoded using a second constrained code, which determines which look-up tables are applied during the first encoding stage. The final codeword, found by cascading the output words of the look-up tables, is transmitted. A decoder can uniquely restore the source data encoded by the first and second codes by observing the received codeword. We demonstrate that the concatenated constrained code enables the design of longer codewords with less redundancy than conventional methods, while avoiding the need for impractically large look-up tables.

We demonstrate the versatility of the concatenated constrained coding scheme through two case studies: designing long constant-weight and low-weight codes.

**Paper structure:** We introduce the fundamentals of concatenated constrained codes in Section 2. Section 3 reviews prior work on the properties of constant-weight codes, which sets a baseline for the new constructions. Section 4 presents new constructions of constant-weight codes using concatenated constrained codes. The construction of very long constant-weight codes is explored in Section 5, where we analyze the application of one concatenated constrained code layered on top of another. Applications to low-weight codes are covered in Section 6. Finally, Section 7 concludes the paper.

## 2. Concatenated Constrained Code, Basics

We describe an encoder that translates binary source data into *n*-bit binary codewords, denoted by ***y***, which satisfy a given constraint A. Let $S_{A}$ denote the set of *n*-bit codewords ***y*** that meet constraint A. Since *n* is typically too large to permit direct generation of codewords via a single look-up table, the codeword ***y*** is divided into *k* equal-sized *m*-bit constrained subwords $y_{t}\in {\left\{0,1\right\}}^{m}$ and $y=(y_{1},y_{2},\ldots ,y_{k})$ and n=km. The parameter *m* is chosen to be small enough to allow the use of look-up tables for mapping source data to the subwords $y_{t}$.

### 2.1. Major Components Overview

We construct *K*, K≤k, distinct sets of *m*-bit subwords, denoted by $S_{i}\subset {\left\{0,1\right\}}^{m}$, 0≤i≤K−1, each satisfying a specific constraint $A_{i}$, 0≤i≤K−1. In practice, only K≤k distinct look-up tables are required as they can be re-used via multiplexing. Each set $S_{i}$ enables a bijective mapping $f_{i}:{\left\{0,1\right\}}^{\nu_{i}}\to S_{i}$ of $\nu_{i}\leq m$ source bits into valid subwords, ensuring compatibility with $A_{i}$.

To construct an *n*-bit codeword ***y***, we concatenate *k m*-bit subwords $y_{t}$, where each $y_{t}$ is selected from the corresponding subword set $S_{c_{t}}$. The selection is guided by a control code $c=(c_{1},\ldots ,c_{k})$, with $c_{t}\in \left\{0,\ldots ,K-1\right\}$. The goal is to ensure that the full codeword $y=(y_{1},\ldots ,y_{k})$ satisfies the overall constraint A.

### 2.2. Basic Properties

The concatenated constrained coding scheme adheres to the following conditions:

- The sets $S_{i}$, 0≤i≤K−1, must be pairwise disjoint.
- Each codeword ***y*** must encode a fixed number of source bits.
- Every codeword ***y*** must satisfy the global constraint A.

We define two segments of source data:

- $a=(a_{1},\ldots ,a_{\ell })$, $a_{t}\in \left\{0,1\right\}$.
- $\hat{a}=\left({\hat{a}}_{1},\ldots ,{\hat{a}}_{\ell_{c}}\right)$, ${\hat{a}}_{t}\in \left\{0,1\right\}$.

The total number of encoded source bits, $\ell +\ell_{c}$, is referred to as the throughput of the code.

The encoder generates the control word $c=(c_{1},\ldots ,c_{k})$, $c_{t}\in \left\{0,\ldots ,K-1\right\}$, by applying a bijective mapping $g:{\left\{0,1\right\}}^{\ell_{c}}\to C$, where *C* is a constrained code. The *ℓ*-bit source data, ***a***, are partitioned into *k* segments $a_{t}$ of $\nu_{c_{t}}$ bits, 1≤t≤k. Clearly, we demand $(a_{1},a_{2},\ldots ,a_{k})=a$ and thus $\sum_{i=1}^{k}\nu_{c_{i}}=\ell$. The set of allowed vectors ***c*** is

(1) $$\begin{array}{c} C=\{c\in {\left\{0,\ldots ,K-1\right\}}^{k}:\\ \left(f_{c_{1}}\left(a_{1}\right),f_{c_{2}}\left(a_{2}\right),\ldots ,f_{c_{k}}\left(a_{k}\right)\right)\in S_{A}\wedge \sum_{i=1}^{k}\nu_{c_{i}}=\ell \}. \end{array}$$

This allows up to $\ell_{c}=\lfloor \log_{2}\left|C|\right\rfloor$ additional source bits to be encoded into the control vector ***c*** via the mapping g(·). Consequently, the total number of source bits that can be represented in the *n*-bit codeword ***y*** is $\ell +\ell_{c}$. Each of the *k* segments of the original source data, denoted $a_{t}$, is mapped to an *m*-bit constrained subword $y_{t}$ using the mapping $y_{t}=f_{c_{t}}\left(a_{t}\right)$ for 1≤t≤k. These subwords are then concatenated to form the complete codeword ***y***, which is subsequently transmitted or stored. Importantly, the control vector ***c*** is not transmitted to the receiver. However, since the subword sets $S_{i}$ are pairwise disjoint, the decoder can uniquely identify which mapping was used for each $y_{t}$. This ensures that both parts of the source data, ***a*** and $\hat{a}$, can be fully and uniquely reconstructed from the received codeword ***y***.

**Figure 1:**
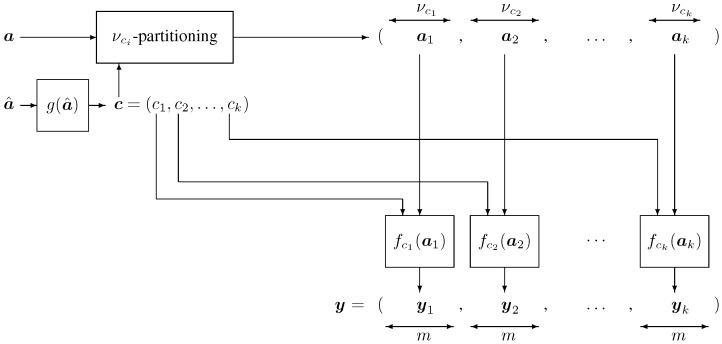
Basic block diagram of a concatenated constrained code. The source data are partitioned into two parts, denoted by $\hat{a}$ and ***a***. The portion $\hat{a}$ is mapped to a constrained codeword ***c*** by the mapping $c=g(\hat{a})$. The remaining source data ***a*** are partitioned, under the control of ***c***, into *k* segments $a_{t}$ of length $\nu_{c_{t}}$, t=1,2,…,k, and are subsequently translated into the final codeword $y=(y_{1},\ldots ,y_{k})$ using functions $y_{t}=f_{c_{t}}\left(a_{t}\right)$ for t=1,2,…,k. The composite codeword ***y*** is sent to the receiver while the auxiliary word *c* is discarded. Although the block diagram shows *k* separate look-up tables for conceptual clarity, in practice only K<k distinct look-up tables are required, as they can be reused via multiplexing.

**Figure 2:**
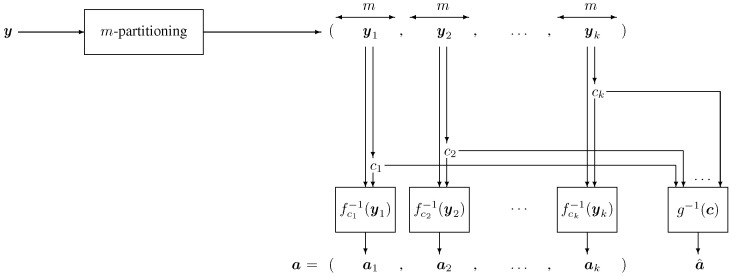
Block diagram of a decoder for a concatenated constrained code. The received vector ***y***, of length n=km, is first partitioned into *k* subwords $y_{i}$, each of length *m*. Using the appropriate inverse look-up tables, the decoder recovers the control vector ***c*** and the data segments $a_{t}$ via the relation $a_{t}=f{c_{t}}^{-1}\left(y_{t}\right)$ for t=1,…,k. Subsequently, the remaining source data $\hat{a}$ are reconstructed by applying the inverse mapping $\hat{a}=g^{-1}\left(c\right)$.

Figure 1 and Figure 2 depict a block diagram of the encoder and decoder, respectively.

### 2.3. Complexity Issues

The encoder begins by mapping a segment of $\ell_{c}$ source bits into a control codeword ***c*** using the bijective function $c=g(\hat{a})$. It then proceeds to convert each of the *k* source subwords $a_{t}$, each of length $\nu_{c_{t}}$ for t=1,2,…,k, into corresponding *m*-bit constrained subwords $y_{t}$ via the mapping $y_{t}=f_{c_{t}}\left(a_{t}\right)$.

Assuming the *k* conversions $y_{t}=f_{c_{t}}\left(a_{t}\right)$ can be implemented in a multiplexed fashion, the primary hardware requirements for encoding and decoding a concatenated constrained code are limited to the following:

- The *K* look-up tables for the sets $S_{i}$, where 0≤i≤K−1.
- A look-up table for the mapping function g(·) that generates *c*.

Unlike traditional approaches that rely on a single large *n*-bit look-up table, the maximum codeword length *n* achievable with the proposed concatenated constrained coding scheme is governed by the *product* of the practical maximum sizes of the *K* look-up tables $S_{i}$ and the control codebook *C*. This significantly relaxes the constraints on memory size and look-up complexity.

A compelling example of this technique is the design of a binary constant-weight code, where each n=128-bit codeword contains exactly 32 ones. This design is efficiently realizable using just three modest-sized look-up tables, highlighting the practicality of the concatenated constrained coding approach.

**Example** **1.** *We design an encoder that converts arbitrary source data into binary codewords, **y**, $y_{i}\in \left\{0,1\right\}$, of length n = 128, where each codeword has 32 1’s and 96 0’s. The maximum number of source bits that can be accommodated is ⌊log212832⌋=100, where ⌊·⌋ denotes the truncation or floor function. Therefore, any code constructed for this setting must have a redundancy of at least 28 bits. Since n = 128 is too large to be handled by a single look-up table, we partition the codeword into k=16 subwords, each of length m = 8 bits. While other values for k and m are possible, the chosen values simplify manual calculations. In the prior art block code design of multiply constant-weight codes [16] or constant subblock-composition code [17], each subword contains exactly two 1’s, and the k=16 subwords can be freely cascaded with each other to form a codeword. Clearly, there are 82=28 possible 8-bit subwords. Twelve excess subwords are deleted, so that each subword can convey four source bits, and hence, the 128-bit codewords can carry 16×4=64 source bits, resulting in 64 bits of redundancy.*

*Using the concatenated constrained code construction, we define K=2 types of subword sets, namely the set of 8-bit words with a single 1, that is*

$$S_{0}=\left\{x\in {\left\{0,1\right\}}^{8}:\sum_{i=1}^{8}x_{i}=1\right\}$$

*and the set of 8-bit words with three 1’s, namely*

$$S_{1}=\left\{x\in {\left\{0,1\right\}}^{8}:\sum_{i=1}^{8}x_{i}=3\right\}.$$

*We easily find that |S0|=81=8 and |S1|=83=56, where |X| denotes the cardinality of the set X. We delete 56−32=24 excess subwords from $S_{1}$ (keeping 32 subwords). Consequently, we have $\nu_{0}=3$ and $\nu_{1}=5$. Define the bijective mappings $f_{0}:{\left\{0,1\right\}}^{3}\to S_{0}$ and $f_{1}:{\left\{0,1\right\}}^{5}\to S_{1}$. Each 128-bit codeword contains 32 ones by selecting eight subwords from $S_{0}$ and eight from $S_{1}$. The k=16 subwords together convey ℓ=8×(3+5)=64 source bits, matching the number of source bits in the prior art; so nothing is gained or lost so far. The k(=16)-bit word, **c**, whose elements determine whether to apply $f_{0}$ or $f_{1}$, has equal numbers of 1’s and 0’s. The word, **c**, can therefore convey ℓc=⌊log2168⌋=13 source bits. As a result, the total, combined, throughput is $\ell +\ell_{c}=64+13=77$.*

*Alternatively, we may choose m=16 and k=8, which requires larger look-up table for $S_{0}$ and $S_{1}$. In this case, the traditional approach yields a throughput of 8×10=80 bits. In contrast, the concatenated constrained code construction achieves a throughput of 4(9+12)+6=90 bits, that is, 90% of the maximum possible.*

The above example shows that the new scheme leverages both subword composition and control-word combinatorics to improve data throughput beyond traditional designs.

In the next sections, we exploit the concatenated constrained code for the construction of efficient constant-weight and low-weight codes. We start with a review of the state of the art.

## 3. Constant-Weight Codes, Preliminaries, Redundancy, Prior Art

### 3.1. Introduction

In a binary constant-weight code, each codeword has the same number of 1’s. These codes, also known as ‘*m*-out-of-*n*’ [7], codes, have found applications across a wide range of devices and systems including data storage [2], code-division multiple-access (CDMA) systems for optical fibers [18], test pattern generation for circuit testing [19], identification coding [20], and VLSI design [21,22]. Traditionally, constant-weight codes have been employed in data transmission and data storage systems that suffer from low-frequency noise or are subject to low-frequency bandwidth constraints [2,23,24]. More recently, they have been proposed for use in DNA-based storage systems. Knuth’s celebrated implementation [25,26] generates ‘balanced’ codewords i.e., codewords of weight $w=\frac{n}{2}$, *n* even, with a computational complexity that scales with *n*, and a redundancy that is approximately twice the theoretical minimum for large *n*.

A simple and efficient algorithm for producing a codeword with a prescribed imbalance is not available, and the development of such an algorithm remains an open research problem [22,27]. Designing low-complexity encoder/decoder architectures for very high-rate constant-weight codes is of significant practical and economic value. There is a clear demand for efficient constant-weight codes that avoid the need of exorbitantly large look-up tables while maintaining high performance.

### 3.2. Redundancy

Let $x=(x_{1},\ldots ,x_{n})$, $x_{i}\in \left\{0,1\right\}$, be a binary word of length *n*. The *Hamming weight* of ***x***, denoted by w(x), is defined by $w\left(x\right)=\sum_{i=1}^{n}x_{i}$. A constant-weight code, denoted by S(w,n), defined by

(2) $$S\left(w,n\right)=\left\{x\in {\left\{0,1\right\}}^{n}:w\left(x\right)=w\right\},$$

comprises all binary words of length *n* and weight *w*, 0≤w≤n. The size of S(w,n), denoted by |S(w,n)|, equals

(3) |S(w,n)|=nγn,

where $\gamma =\frac{w}{n}$, $0\leq \gamma \leq \frac{1}{2}$, denotes the *relative weight*. In case we have a code with codewords with equal numbers of 1’s and 0’s, i.e., 2w=n, *n* even, the code is said to be *balanced*. Note that without loss of generality we study the case γ≤1/2 as the case γ>1/2 is simply found by inverting (flipping) the binary symbols of a codeword.

The *capacity* and *maxentropic* redundancy of constant-weight codes, denoted by C(w,n) and ρ(w,n), are defined by

(4) C(w,n)=log2|S(w,n)|=log2nγn

and

(5) ρ(w,n)=n−C(w,n).

Using [28], we find the useful approximation

(6) $$\rho \left(w,n\right)\approx \frac{1}{2}\log_{2}n+\left(1-H(\gamma )\right)n+\frac{1}{2}\log_{2}\frac{\pi }{2},\;n\gg 1,$$

where Shannon’s *entropy* function, H(x), is defined by

(7) $$H\left(x\right)=-x\log_{2}\left(x\right)-\left(1-x\right)\log_{2}\left(1-x\right).$$

The maxentropic redundancy, ρ(w,n), is applied as a yardstick to evaluate the performance of implemented codes.

### 3.3. Traditional Code Design Approach

In the prior art, see [29] and the references therein, a codeword, ***y***, of length *n* is divided into *k* constrained subwords, $y_{i}$, 1≤i≤k, of equal length *m*, where n=km. The weight of each subword, $y_{i}$, is prescribed and denoted by $\omega_{i}=w\left(y_{i}\right)$, $\omega_{i}\in \left\{0,\ldots ,m\right\}$, where $\sum_{i=1}^{k}\omega_{i}=w$. The weight distribution vector is denoted by $\omega =(\omega_{1},\ldots ,\omega_{k})$. The redundancy, denoted by $\rho_{1}\left(w,n\right)$, equals, see (5),

(8) $$\rho_{1}\left(w,n\right)=\sum_{i=1}^{k}\rho \left(\omega_{i},m\right).$$

Note that $\rho_{1}\left(w,n\right)$ describes a lower bound to the redundancy of implemented codes as in practice the (sub)code sizes mωi are truncated to the nearest power of two, see Example 1 for an illustration. We have opted not to truncate the code sizes in our computations, except in the worked design examples, in order to maintain analytical tractability. The *efficiency* of this construction, denoted by $\eta_{1}$, is defined as [29]

(9) $$\eta_{1}=\frac{\rho (w,n)}{\rho_{1}\left(w,n\right)}.$$

It is shown in the Appendix A that a uniform, or *flat*, weight distribution minimizes the redundancy.

Numerical results for the efficiency of the prior art construction, $\eta_{1}$, are presented in Table 1. Additional efficiency parameters, $\eta_{2}$ and $\eta_{3}$, for the new constructions are introduced and discussed in Section 4.2 and Section 4.3, respectively.

In the next section, we apply the concatenated constrained code to the design of constant-weight codes.

## 4. Concatenated Constant-Weight Code

### 4.1. Concatenated Constrained Code

As in Section 3.3, the *n*-bit codeword ***y*** is partitioned into *k* subwords, $y_{i}$, 1≤i≤k, of equal length *m*. We have $w\left(y_{i}\right)=\omega_{i}$, $\omega_{i}\in \left\{0,\ldots ,m\right\}$ and $\sum_{i=1}^{k}\omega_{i}=w$. In the prior art, the subword weights, $\omega_{i}=w\left(y_{i}\right)$, are assumed to be fixed for the whole transmission. In this section, however, the weights of the subwords are controlled, ‘modulated’ in engineering terms, by a constrained codeword $\omega =(\omega_{1},\ldots ,\omega_{k})$, which is taken from the predefined set Ω. Note that ω and Ω play the same role as ***c*** and *C* in Section 2.

The coding of ***y*** is carried out in two distinct steps. First, source data are mapped bijectively to the weight distribution vector ω∈Ω via a look-up table. In the second step, the encoder uses the look-up tables corresponding to the constant-weight codes $S(\omega_{i},m)$, 1≤i≤k, to convert the source data into the subwords $y_{i}$. Since the mapping $\omega_{i}$ to $S(\omega_{i},m)$, 1≤i≤k, is bijective, the decoder can uniquely determine the vector ω, and thereby recover the original source data from the received ***y***.

Define the vector $u=(u_{0},u_{1},\ldots ,u_{m})$, $u_{j}\in \left\{0,\ldots ,k\right\}$, where $u_{j}$ represents the number of occurrences of the symbol *j* in ω. The vector ***u*** is commonly referred to as the *histogram* of ω. An allowed vector ***u*** must satisfy

(10) $$\sum_{i=0}^{m}u_{i}=k\;\mathrm{and}\sum_{i=0}^{m}iu_{i}=w.$$

The set Ω is the set of *k*-vectors, ω, of fixed composition $\left(u_{0},u_{1},\ldots ,u_{m}\right)$, that is, the number of 0’s, 1’s, …,m’s in ω is given by $u_{0},u_{1},\ldots ,u_{m}$, respectively. The size of the constant composition code Ω is

(11) $$\left|\Omega \right|=\frac{k!}{u_{0}!u_{1}!\ldots u_{m}!}.$$

The number of source words that can be accommodated, denoted by $M_{\Omega }$, equals

(12) MΩ=k!u0!u1!…um!∏i=0mmiui.

Below we illustrate and analyze a design of two simple cases, where ***u*** contains K=2 or K=3 non-zero elements, which are denoted here by the binary and ternary case. We start with a description of the binary case, K=2.

### 4.2. Binary Case, K=2

Let $\omega_{i}\in \left\{v_{0},v_{1}\right\}$, so that $u_{v_{0}}\neq 0$ and $u_{v_{1}}\neq 0$, and otherwise $u_{i}=0$. We obtain from (10) that

(13) $$u_{v_{0}}+u_{v_{1}}=k\;\mathrm{and}v_{0}u_{v_{0}}+v_{1}u_{v_{1}}=w.$$

There are manifold solutions to the above system of (positive) integer equations. An enticing option for *k* even is $v_{0}=v-1$ and $v_{1}=v+1$, so that $u_{v-1}=u_{v+1}=\frac{k}{2}$, and

(14) $$\Omega_{2}=\left\{\omega \in {\left\{v-1,v+1\right\}}^{k}:\sum_{i=1}^{k}\omega_{i}=w\right\}$$

denotes the set of allowed vectors ω.

The relationship between ω and ***c*** is a straightforward renumbering of their elements by

(15) $$c_{i}=\left(\omega_{i}-v+1\right)/2,\;\;\;i=1,\ldots ,k,$$

so that $c_{i}\in \left\{0,1\right\}$, and renaming $S_{0}=S\left(v-1,m\right)$ and $S_{1}=S\left(v+1,m\right)$. The coding circuitry comprises two look-up tables, $S_{0}$ and $S_{1}$, of width *m*, plus a (binary) look-up table for the balanced code, *C*, of width *k*. Note that Knuth’s algorithm can be used for mounting *k*, when the look-up table for *c* is uneconomically expensive.

We obtain from (14) that

(16) |Ω2|=kk2,

so that, see (12),

(17) MΩ2=|S(v−1,m)|k2|S(v+1,m)|k2kk2.

Hence the redundancy, denoted by $\rho_{2}\left(w,n\right)$, is

ρ2(w,n)=n−k2log2mv−1mv+1−log2kk2.

Since

(18) mv−1=vm−v+1mv

and

(19) mv+1=m−vv+1mv,

we obtain

(20) mv−1mv+1=v(m−v)(v+1)(m−v+1)mv2,

so that

(21) ρ2(w,n)=kρ(v,m)+β(v,m)−1klog2kk2=ρ1(v,m)+kβ(v,m)−log2kk2,

where

(22) $$\beta \left(v,m\right)=-\frac{1}{2}\log_{2}\frac{v(m-v)}{\left(v+1)(m-v+1\right)}.$$

**Figure 3:**
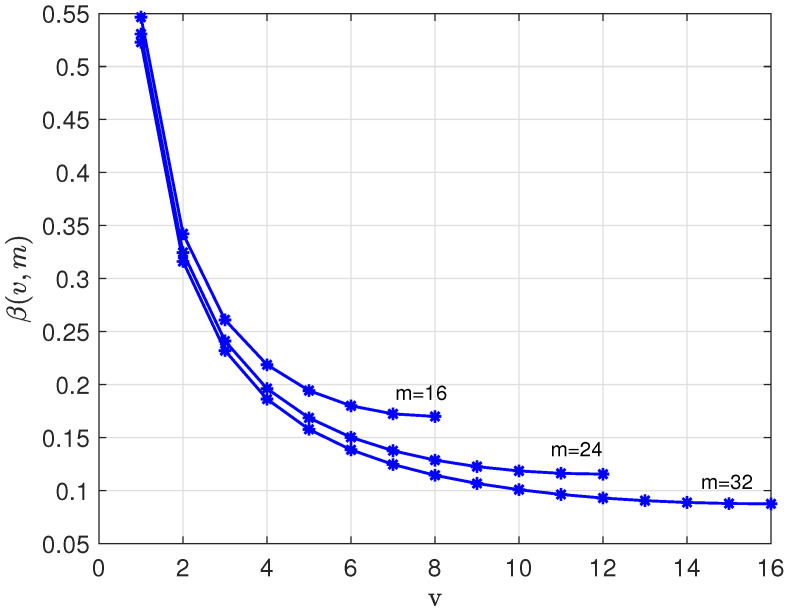
Coefficient β(v,m) versus *v* for m=16, 24, and 32.

Figure 3 shows the coefficient β(v,m) versus *v*, 1≤v≤m/2, for m=16,24, and 32.

The coefficient β(v,m) is an important parameter as it quantifies the additional redundancy per subword required for the concatenated coding system. Note in (21) that, with respect to the redundancy of the traditional code, $\rho_{1}\left(w,n\right)$, we have two additional terms in $\rho_{2}\left(w,n\right)$. On one hand we add redundancy, namely β(v,m) bit per subword, by deviating from the flat weight distribution, as discussed in Appendix A. On the other hand, we reduce redundancy, namely

(23) 1klog2kk2≈1−12klog2(k)

bits per subword by encoding data in the second (binary) code *C*. Figure 3 shows that the loss, β(v,m), is around 0.1–0.2 bits per subword over a wide range of the parameters *v* and *m*.

The efficiency of codes based on the binary concatenated constrained code is defined by

(24) $$\eta_{2}=\frac{\rho (w,n)}{\rho_{2}\left(w,n\right)}.$$

Table 1 presents numerical results for the code’s efficiency, $\eta_{2}$. We observe a notable improvement in efficiency compared to that of the traditional scheme, $\eta_{1}$, especially for larger values of *k*.

### 4.3. Ternary Case, K=3

For the ternary case, we define the values of $u_{i}$ by

(25) $$u_{v-1}=u_{v+1}=\frac{k-a}{2}\;\mathrm{and}\;u_{v}=a,\;a=2,4,\ldots ,k-2$$

and otherwise $u_{i}=0$. Note that for a=0 we have the binary case, K=2, as detailed in the previous subsection. Let $\Omega_{3}$ denote the constant composition code based on ***u***. Then

(26) $$|\Omega_{3}|=\frac{k!}{\frac{k-a}{2}!a!\frac{k-a}{2}!},$$

and the number of source words that can be accommodated, denoted by $M_{\Omega_{3}}$, equals

(27) $$M_{\Omega_{3}}={\left|S\left(v-1,m\right)\right|}^{\frac{k-a}{2}}{\left|S\left(v,m\right)\right|}^{a}{\left|S\left(v+1,m\right)\right|}^{\frac{k-a}{2}}\left|\Omega_{3}\right|.$$

The overall redundancy, denoted by $\rho_{3}\left(w,n\right)$, is

(28) $$\rho_{3}\left(w,n\right)=k\rho \left(v,m\right)+\left(k-a\right)\beta \left(v,m\right))-\log_{2}\left|\Omega_{3}\right|.$$

For the special case $a=\frac{k}{3}$, kmod3=0, we have

(29) $$|\Omega_{3}|=\frac{k!}{{\left(\frac{k}{3}!\right)}^{3}}$$

and

(30) $$\rho_{3}\left(w,n\right)=k\left(\rho \left(v,m\right)+\frac{2}{3}\beta \left(v,m\right)\right)-\log_{2}\frac{k!}{{\left(\frac{k}{3}!\right)}^{3}}.$$

For k≫1, we obtain the Stirling approximation [30]

(31) $$\frac{k!}{{\left(\frac{k}{3}!\right)}^{3}}\approx \frac{3^{k+\frac{3}{2}}}{2\pi k},\;\;k\gg 1,$$

so that

(32) $$\rho_{3}\left(w,n\right)\approx k\left(\rho \left(v,m\right)+\frac{2}{3}\beta \left(v,m\right)-\log_{2}\left(3\right)\right).$$

Define the efficiency of this construction by

(33) $$\eta_{3}=\frac{\rho (w,n)}{\rho_{3}\left(w,n\right)}.$$

Table 1 shows numerical results for the code’s efficiency, $\eta_{3}$. As in the binary case, we find ***c*** by renumbering the elements of ω according to $c_{i}=\omega_{i}-v+1$, i=1,…,k, so that $c_{i}\in \left\{0,1,2\right\}$. We then rename the sets as $S_{0}=S\left(v-1,m\right)$, $S_{1}=S\left(v,m\right)$ and $S_{2}=S\left(v+1,m\right)$. It is assumed that a look-up table is used to map the source data $\hat{a}$ into ***c***. For larger values of *k*, where using a look-up table becomes impractical, we may resort to enumeration algorithms for multiset permutations [31] or Knuth-like algorithms [30,32], which can be employed instead. The next example elaborates Example 1 for K=3.

**Example** **2.** *We choose, as in Example 1, n=128, w=32, v=2, and m=8. For the ternary case, K=3, we obtain the following results from Example 1: $\lfloor \log_{2}\left(|S\left(1,8\right)|)\right\rfloor =3$, $\lfloor \log_{2}\left(|S\left(2,8\right)|)\right\rfloor =4$, and $\lfloor \log_{2}\left(|S\left(3,8\right)|)\right\rfloor =5$. From this, we can easily determine that the maximum throughput $\ell +\ell_{c}=84$ is attained for u=(0,6,4,6,0,0,0,0,0) or u=(0,5,6,5,0,0,0,0,0).*

### 4.4. Error Propagation Effects

Single bit errors in the received codeword can lead to an avalanche of errors during decoding. This phenomenon, called *error propagation,* is especially pronounced in long codes based on enumerative methods [14]. In this subsection, we examine the error propagation behavior of constant-weight codes constructed using concatenated constrained codes.

Let the received codeword be denoted by $\hat{y}=\left({\hat{y}}_{1},\ldots ,{\hat{y}}_{k}\right)$, which differs from the transmitted codeword, $y=(y_{1},\ldots ,y_{k})$, in a single (unknown) position. Since ***y*** belongs to a constant-weight code, such an error is detectable.

For a constant-weight code constructed with parameter K=2 (binary case), as illustrated in Example 1, each subword $y_{i}$ belongs to the set S(v−1,m)∪S(v+1,m), 1≤i≤k, and v=w/k. Since the minimum Hamming distance between elements of this set is two, it is possible to identify the specific subword $y_{i}$ that was received in error. Assume that the subword ${\hat{y}}_{j}$ with index *j* is in error, i.e., ${\hat{y}}_{j}\neq y_{j}$. By definition, we have $\sum_{i=1}^{k}c_{i}=k/2$, so the control vector *c* can be fully decoded by, see (15),

(34) $$c_{i}=\left\{\begin{array}{cc} \frac{\left(w\left({\hat{y}}_{i}\right)-v+1\right)}{2}, & 1\leq i\leq k,i\neq j\\ \frac{k}{2}-\sum_{i=1,i\neq j}^{k}c_{i}, & i=j. \end{array}\right.$$

Thus, in the binary case, K=2, all data can be recovered except for the unknown *m*-bit subword ${\hat{y}}_{j}$, which must be marked as an erasure. In the ternary case (K=3), each $y_{i}$ belongs to the set S(v−1,m)∪S(v,m)∪S(v+1,m), 1≤i≤k. Since the minimum Hamming distance between its elements is one, it is not always possible to identify the erroneous subword. As a result, the entire *n*-bit codeword must be flagged as an erasure. Note that for the ternary case we may choose the sets S(v−2,m), S(v,m) and S(v+2,m), so that it is also possible to identify the specific subword $y_{i}$ that was received in error.

## 5. Very Long Codes

In the previous sections, we demonstrate that concatenated constrained coding can efficiently produce longer constant-weight codewords compared to conventional state-of-the-art methods. However, the scalability of such codes is limited: the codeword length n=km is, in practice, constrained by the maximum feasible size of the look-up tables, either the subword tables $S_{i}$ of width *m*, or the control code table *C* of width *k*.

To overcome these practical limitations, several strategies can be adopted:

- Use enumerative coding techniques to generate the subword sets $S_{i}$ and/or the control codebook *C*.
- Apply a second layer of concatenated constrained coding to generate entries within $S_{i}$ and/or *C*, effectively building a hierarchy of constrained encodings.

The next example illustrates the effectiveness of this method through numerical results for a constant-weight code of length n=2048.

**Example** **3.** *Consider the case where n=2048 and w=512 (γ=1/4). The theoretical minimum redundancy for this constant-weight code is ρ(512,2048)=392.12 bits. Due to the large value of n, enumerative coding becomes impractical for this low-weight scenario, so we explore alternative coding strategies:*

- *We can cascade sixteen codewords of length $n^{\prime }=128$ and $w^{\prime }=32$, see Example 2, with a total throughput of 16×84=1344 bit.*
- *We can, as illustrated in Example 1, select a concatenated constrained code with m=8 and k=256. Since k=256 is too large for directly using a look-up table to generate the control word **c**, we alternatively consider applying enumerative coding for generating the balanced codeword **c**. Then, the redundancy is five bit, so that the scheme’s throughput is 256−5+k×4=1275 bit. We may apply Knuth’s algorithm to generate codeword **c** of length k=256 with an 8-bit redundancy, so that the throughput is 256−8+k×4=1272 bit.*
- *We may construct a long code by using multiple smaller concatenated constrained codes as building blocks. For example, an (n=2048, w=512) code is constructed by first constructing two concatenated constrained codes with parameters $n^{\prime }=128$ of weights $w^{\prime }=31$ and w″=33, respectively, by using $u^{\prime }=\left(0,6,5,5,0,0,0,0,0\right)$ and u″=(0,5,5,6,0,0,0,0,0) instead of u=(0,6,4,6,0,0,0,0,0) of the $n^{\prime }=128$, $w^{\prime }=32$ code detailed in Example 2. Both codes have a throughput of 84 bit. On top of these codes, we define a concatenated constrained code with k=16 and $n^{\prime }=128$, the combined throughput equals 16×84+20=1364 bit. The code’s implementation requires six small look-up tables.*

*While numerous other design options exist beyond those discussed above, the examples provided offer a representative insight into the key trade-offs involved.*

## 6. Low-Weight Codes

Low-weight, or sometimes referred to as light-weight or bounded-weight, codes have found applications in efficiently synthesizing deoxyribonucleic acid (DNA) for massive data storage, where multiple DNA strands are synthesized in parallel [33]. Applications can also be found in memristor crossbar arrays for reducing the number of sneak-paths [34], and simultaneous energy and data transfer [17,35].

A low-weight code of length *n* and weight at most *t*, 0≤t≤n, denoted by $\hat{S}\left(t,n\right)$, is defined by the union of the sets of words of weight w≤t,

(35) $$\hat{S}\left(t,n\right)=\overset{t}{\underset{w=0}{⋃}}S\left(w,n\right).$$

The redundancy, denoted by $\hat{\rho }\left(t,n\right)$, equals

(36) ρ^(t,n)=n−log2|S^(t,n)|=n−log2∑w=0tnw.

For asymptotically large *n* we obtain [28]

(37) $$\frac{1}{n}\hat{\rho }\left(t,n\right)\approx 1-H\left(\frac{t}{n}\right),\;\;\frac{t}{n}<\frac{1}{2}.$$

**Figure 4:**
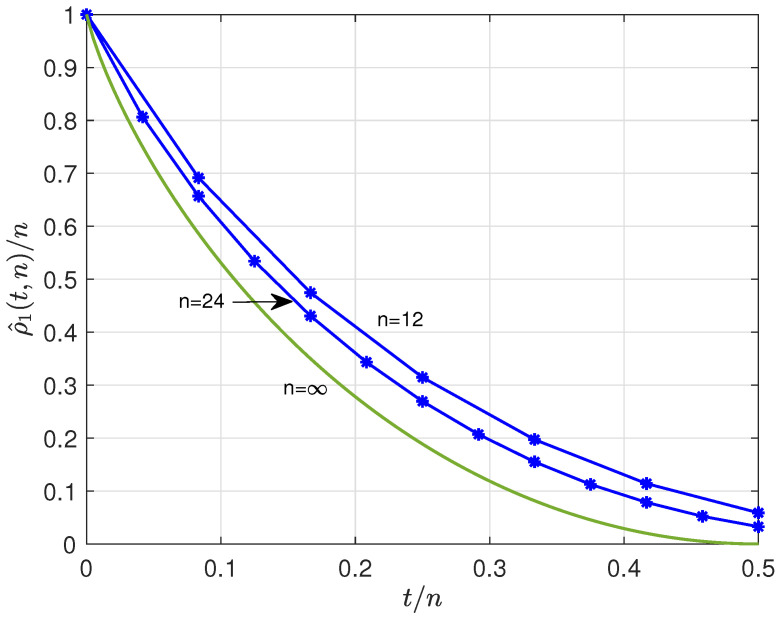
Lower bound normalized redundancy, $\hat{\rho }\left(t,n\right)/n$, of low-weight codes versus t/n for n=12, 24, and *∞*.

Figure 4 shows the normalized redundancy, $\hat{\rho }\left(t,n\right)/n$, versus relative maximum weight, t/n for n=12,24, and *∞*.

### 6.1. Code Design

The conventional construction of a low-weight block code follows a similar approach to that of constant-weight codes. The source data and codewords are partitioned into *k* manageable subwords, with look-up tables used to map the source data into these subwords. As assumed above let n=km be the length of the low-weight codeword, where *m* is the length of each subword, and t=kv. The redundancy of a conventional code, denoted by ${\hat{\rho }}_{1}\left(t,n\right)$, is

(38) $${\hat{\rho }}_{1}\left(t,n\right)=k\hat{\rho }\left(v,m\right).$$

In the next subsection, we describe a simple low-weight code based on the concatenated constrained code format.

#### Binary Case, K=2

The source data are divided into two segments, namely $\hat{a}$ and ***a***. The first segment, $\hat{a}$, is translated into a binary constrained codeword, $c=\left(c_{1},\ldots ,c_{k}\right)=g\left(\hat{a}\right)$, where $c_{i}\in \left\{0,1\right\}$, of length *k*. The second segment, ***a***, is translated into a series of *k* weight-constrained *m*-bit words, each taken from either the set $S_{0}\left(v,m\right)$ or $S_{1}\left(v,m\right)$. For a concatenated constrained coding construction, the sets $S_{0}\left(v,m\right)$ and $S_{1}\left(v,m\right)$ must be disjoint. A convenient choice for these sets is

(39) $$S_{0}\left(v,m\right)=S\left(v+1,m\right)\cup S\left(v,m\right)$$

and

(40) $$S_{1}\left(v,m\right)=\overset{v-1}{\underset{w=0}{⋃}}S\left(w,m\right).$$

Let $c=(c_{1},\ldots ,c_{k})$ be a balanced word, *k* even, then the *n*-bit codeword found by cascading *k m*-bit subwords from $S_{c_{i}}\left(v,m\right)$, 1≤i≤k, has a weight less than or equal to *t*. The redundancy of the code, denoted by ${\hat{\rho }}_{2}\left(t,n\right)$, equals

(41) ρ^2(t,n)=n−k2log2|S0(v,m)||S1(v,m)|−log2kk2.

Define

(42) $$\hat{\beta }\left(v,m\right)=-\frac{1}{2}\log_{2}\frac{|S_{0}\left(v,m\right)||S_{1}\left(v,m\right)|}{|\hat{S}{\left(v,m\right)|}^{2}},$$

then

(43) ρ^2(t,n)=k(ρ^(v,m)+β^(v,m))−log2kk2.

**Figure 5:**
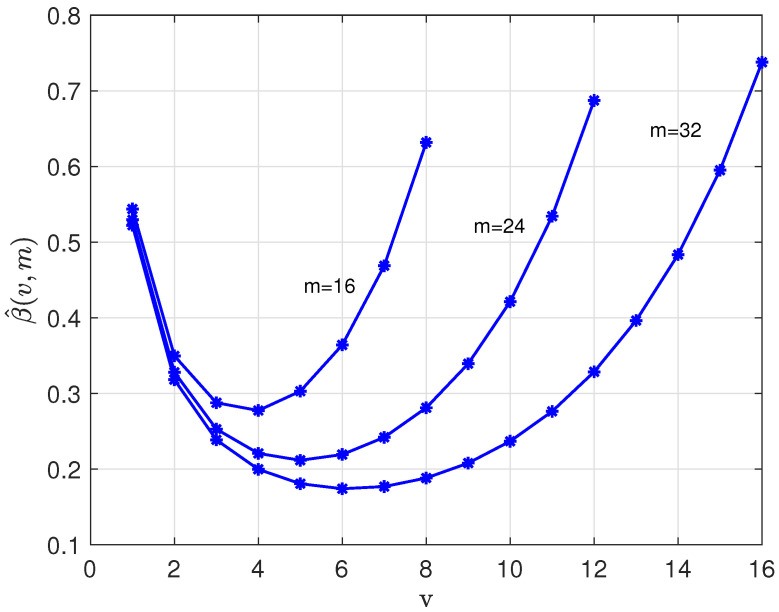
Coefficient $\hat{\beta }\left(v,m\right)$ versus *v* for m=16, 24, and 32.

Figure 5 shows the coefficient $\hat{\beta }\left(v,m\right)$ versus *v*, 1≤v≤m/2, for m=16,24, and 32.

Define the efficiency parameters as ${\hat{\eta }}_{1}=\hat{\rho }\left(t,n\right)/{\hat{\rho }}_{1}\left(t,n\right)$ and ${\hat{\eta }}_{2}=\hat{\rho }\left(t,n\right)/{\hat{\rho }}_{2}\left(t,n\right)$. Numerical results are presented in Table 2. It can be observed that the concatenated codes reduce the redundancy compared to the baseline by 6.5 percentage points for k=128.

## 7. Conclusions

We have introduced a new class of constrained codes, referred to as concatenated constrained codes, which enable the construction of very long constrained codewords with significantly reduced complexity. Similar to traditional methods, the source data are divided into smaller blocks. In the first step, one segment of the source data is encoded using a set of small look-up tables, each corresponding to a disjoint set of valid output sequences. In the second step, another segment of the source data is encoded into a control codeword that determines which look-up table is used for each portion of the first segment.

This layered encoding structure enables the generation of longer codewords with lower redundancy compared to conventional approaches, while eliminating the need for massive look-up tables.

We demonstrated the effectiveness of this approach through two case studies focused on constructing binary constant-weight and light-weight codewords of length *n*, each containing exactly *w* ones, or not more than *w* ones, where w≤n/2. The concatenated constrained codes in these examples achieve lower redundancy than leading state-of-the-art solutions, while requiring only three or four compact look-up tables, highlighting both their efficiency and practicality.

We have shown that extremely long codewords can be constructed by applying a second layer of concatenated constrained coding on top of an initial concatenated constrained scheme, effectively generating elements within the sets $S_{i}$ and/or the codebook *C*.

## Figures and Tables

**Table 1:** Code efficiency, $\eta_{1}$, $\eta_{2}$, and $\eta_{3}$ versus codeword length *n*, weight *w*, and number of subwords *k*, where m=n/k, v=w/k, and γ=w/n. The parameter $\eta_{1}$ refers to the efficiency of the prior art code, see (9), while the parameters $\eta_{2}$ and $\eta_{3}$ refer to the code efficiency of the new constructions, see (24) and (33).

*n*	*w*	*k*	*m*	*v*	γ	$\eta_{1}$	$\eta_{2}$	$\eta_{3}$
900	240	60	15	4	0.2667	0.5528	0.6560	0.7792
900	240	30	30	8	0.2667	0.6742	0.7533	0.8195
900	240	12	75	20	0.2667	0.8165	0.8593	0.8864
900	240	6	150	40	0.2667	0.8966	0.9192	0.9317
600	240	120	5	2	0.4000	0.1109	0.1539	0.3828
600	240	60	10	4	0.4000	0.1629	0.2309	0.3778
600	240	30	20	8	0.4000	0.2436	0.3248	0.4277
600	240	24	25	10	0.4000	0.2771	0.3601	0.4528
600	240	12	50	20	0.4000	0.4066	0.4879	0.5540
600	240	6	100	40	0.4000	0.5705	0.6380	0.6814
300	60	30	10	2	0.2000	0.6473	0.7334	0.8714
300	60	12	25	5	0.2000	0.7842	0.8435	0.8943
300	60	6	50	10	0.2000	0.8715	0.9058	0.9283

**Table 2:** Code efficiency, ${\hat{\eta }}_{1}$ and ${\hat{\eta }}_{2}$ versus number of subwords per codeword, *k*, for codeword length n=1024, and maximum weight t=128.

*k*	m=n/k	v=w/k	${\hat{\eta }}_{1}$	${\hat{\eta }}_{2}$
128	8	1	0.7633	0.8294
64	16	2	0.8283	0.8880
32	32	4	0.8851	0.9246
16	64	8	0.9291	0.9513
8	128	16	0.9600	0.9711

## Data Availability

No new data were created or analyzed in this study.

## Appendix A

We investigate minimizing the redundancy of the basic design approach, see Section 3.3.

**Theorem** **A1.**
*The number of words*

M1=∏i=1kmωi

*is maximized by choosing a flat distribution for ω, where the subword weights, $\omega_{i}$, are as close to w/k as possible. Specifically, this means that the difference between any pair of weights is at most 1.*

**Proof.** Let $i_{1}$ and $i_{2}$, $i_{1}\neq i_{2}$, be arbitrarily chosen indices such that $1\leq i_{1},i_{2}\leq n$. Then the number of *n*-sequences is

(A1) M1=mωi1mωi2∏i=1,i≠i1,i2kmωi.

Suppose $\omega_{i_{1}}>\omega_{i_{2}}+1$. To increase $M_{1}$ replace $\omega_{i_{1}}$ by $\omega_{i_{1}}-1$ and $\omega_{i_{2}}$ by $\omega_{i_{2}}+1$ (noting that the sum $\sum_{i=1}^{k}\omega_{i}=w$ remains constant). Then

(A2) M1=mωi1−1mωi2+1∏i=1,i≠i1,i2kmωi,=ωi1m−ωi1+1m−ωi2ωi2+1∏i=1kmωi.

Since $\omega_{i_{1}}>\omega_{i_{2}}+1$, it follows that

(A3) $$\frac{\omega_{i_{1}}}{m-\omega_{i_{1}}+1}\frac{m-\omega_{i_{2}}}{\omega_{i_{2}}+1}>1.$$

Because $i_{1}$ and $i_{2}$ were arbitrary, this argument applies to any pair of indices. Therefore, the number of words $M_{1}$ is maximized when the weights $\omega_{i}$ are balanced as evenly as possible, i.e., when the difference between any two $\omega_{i}$ is at most 1. □
